# Preventive Effects of Cocoa and Cocoa Antioxidants in Colon Cancer

**DOI:** 10.3390/diseases4010006

**Published:** 2016-01-22

**Authors:** María Angeles Martín, Luis Goya, Sonia Ramos

**Affiliations:** Department of Metabolism and Nutrition, Institute of Food Science and Technology and Nutrition (ICTAN-CSIC), José Antonio Novais 10, Ciudad Universitaria, 28040 Madrid, Spain; amartina@ictan.csic.es (M.A.M.); luisgoya@ictan.csic.es (L.G.)

**Keywords:** cocoa flavonoids, colon cancer, *in vitro* and *in vivo* studies, molecular mechanism

## Abstract

Colorectal cancer is one of the main causes of cancer-related mortality in the developed world. Carcinogenesis is a multistage process conventionally defined by the initiation, promotion and progression stages. Natural polyphenolic compounds can act as highly effective antioxidant and chemo-preventive agents able to interfere at the three stages of cancer. Cocoa has been demonstrated to counteract oxidative stress and to have a potential capacity to interact with multiple carcinogenic pathways involved in inflammation, proliferation and apoptosis of initiated and malignant cells. Therefore, restriction of oxidative stress and/or prevention or delayed progression of cancer stages by cocoa antioxidant compounds has gained interest as an effective approach in colorectal cancer prevention. In this review, we look over different *in vitro* and *in vivo* studies that have identified potential targets and mechanisms whereby cocoa and their flavonoids could interfere with colonic cancer. In addition, evidence from human studies is also illustrated.

## 1. Introduction

Cancer is a multistage process conventionally defined by three stages: initiation, promotion and progression [1]. Development of colon cancer typically is initiated from normal epithelial cells via aberrant crypts and progressive adenoma stages to carcinomas *in situ* and then metastasis. Along this process, oxidative stress has the potential to affect numerous signaling pathways related to the proliferation of initiated cells and enhanced malignant transformation [2]. In the initiation stage, the generation of reactive oxygen species (ROS) has been involved in DNA damage and in the development of aberrant crypt foci (ACF) [3,4], the earliest identifiable precancerous lesions in colon cancer [5]. Similarly, in the post-initiation/promotion stages, ROS also contribute to abnormal gene expression and modification of second-messenger systems in epithelial cells within the ACF. In this manner, several signaling pathways involved in cell survival are deregulated, resulting in an increase in cell proliferation and/or a decrease in the apoptosis of the initiated cell population [5]. Therefore, inhibition of oxidative stress together with the modulation of signaling routes related to cell survival/proliferation exerted by natural antioxidant compounds seems to be an effective approach in preventing and slowing down the initiation and progression of colon cancer. In fact, the identification of natural bioactive compounds that downregulate cell proliferation or upregulate apoptotic processes could be a complementary and useful strategy to control the development and progression of colon cancer, and it has become an essential subject for study in current research [6,7]. In this regard, cocoa and its flavonoids have shown other anticarcinogenic properties independently of their conventional antioxidant activity [7,8,9,10]. Consequently, cocoa polyphenols could be considered as promising candidates for colon cancer chemoprevention.

Cocoa, the dried and either unfermented or fermented seeds derived from *Theobroma cacao*, has the highest flavanol content of all foodstuffs on a weight basis and is a significant contributor to the total dietary intake of flavonoids [11,12,13]. In fact, for many individuals, cocoa products constitute a larger proportion of the diet than foodstuffs containing bioactive compounds with similar properties, such as green tea, wine or soy beans [14].

**Figure 1:**
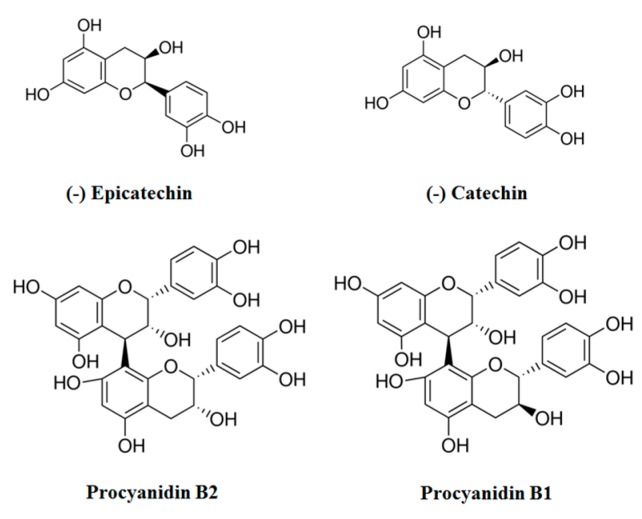
Main flavonoids present in fermented cocoa. Chemical structures of (−)-epicatechin and (−)-catechin and their respective dimmers, procyanidins B2 and B1.

However, health effects derived from cocoa flavonoids depend on their bioavailability, which is also affected by their chemical structure [15]. In this regard, fermented cocoa contains high quantities of flavanols (−)-epicatechin (EC), (−)-catechin and their dimers procyanidins B2 (PB2) and B1 (Figure 1), although other polyphenols, such as naringenin, luteolin, apigenin, quercetin, isoquercitrin (quercetin 3-*O*-glucoside), quercetin 3-*O*-arabinose and hyperoside (quercetin 3-*O*-galactoside), have also been found in minor amounts [16]. Interestingly, in comparison to other flavonoid-containing foodstuffs, cocoa and its derivative products exhibit a high concentration of larger procyanidins that are poorly absorbed through the gut barrier, and consequently, their beneficial effects would be restricted to the gastrointestinal tract, where they may have an important antioxidant and anticarcinogenic local function [6,7,8,9,10]. Thus, those oligomers and polymers of flavanols that are not absorbed in the intestine could be metabolized by the microbiota into low molecular weight phenolic acids, which are more bioavailable, and might be well absorbed through the colon [17,18]. Additionally, recent findings have demonstrated that some of the microbial metabolites derived from cocoa consumption also retain biological properties [19].

Evidence for chemoprevention by bioactive substances is achieved from a combination of epidemiological, animal and basic mechanistic studies. Consequently, the interest for understanding the mode of action of cocoa and its flavanols has been recently rising, especially in cultured colonic cells, although it has not been fully elucidated. In addition, it also remains to be demonstrated whether these mechanisms are involved in cancer prevention in humans. In the present work, different *in vitro* studies that have identified the potential targets and mechanisms whereby cocoa and their polyphenolic compounds could interfere with colonic cancer cells are reviewed. Then, the potential beneficial effects of cocoa in animal models of colon cancer are summarized, and finally, some evidence from human studies is also provided.

## 2. Chemopreventive Effects in Cultured Cells

The effect of cocoa and its main flavanols against colon cancer have been studied in cultured cells, which also allow the elucidation of their molecular mechanisms of action (Table 1). All of these studies have shown that pathways responsible for the potential chemopreventive activity of cocoa and its flavonoids are mainly related to their antioxidant and anti-inflammatory properties and their ability to inhibit proliferation and to induce apoptotic cell death. In addition, to the best of our knowledge, it should be noted that no studies have been performed in HT-29 cells, which is one of the “standard” colon cancer cell models.

**Table 1:** Effects of cocoa and cocoa flavonoids on colonic cancer cultured cell lines ^a^. The arrow indicates an increase (↑) or decrease (↓) in the levels or activity of the different analyzed parameters, and “=” designates an unmodified parameter. DOC, deoxycholic acid.

Biological Activity	Flavonoid	Cell	Concentration	Output	References
Antioxidant	Cocoa	Caco-2	10 µg/mL	acrylamide-incubated cells: ↓ GSH depletion, ↓ ROS generation, ↑ γ-GCS, ↑ GST	[20]
Antioxidant	Procyanidin B2	Caco-2	10 µM (5.79 µg/mL)	acrylamide-incubated cells: ↓GSH depletion, ↓ ROS generation, ↑ γ-GCS, ↑ GST	[20]
Antioxidant	Epicatechin	Caco-2	10 µM (2.9 µg/mL)	acrylamide-incubated cells: ↓ GSH depletion, ↓ ROS generation	[20]
Apoptosis and proliferation/survival	Cocoa	Caco-2	10 µg/mL	↓ acrylamide-induced caspase-3 and p-JNK	[20]
Apoptosis and proliferation/survival	Procyanidin B2	Caco-2	10 µM (5.79 µg/mL)	↑ ERK, ↑ p38, ↓ acrylamide-induced caspase-3 and p-JNK	[20]
Antioxidant	Catechin	Int-407	100 µM (29 µg/mL)	↓ lipid peroxidation, ↓ ROS formation, ↑ GPx, ↑ GR, ↑ Nrf2, ↑ HO-1	[21]
Antioxidant	Epicatechin	Caco-2	1–10 µM (0.29–2.9 µg/mL)	*t*-BOOH-treated cells: ↓ ROS generation, ↓ LDH, = GPx, = GST, = GR	[22]
Apoptosis and proliferation/survival	Procyanidin B2	Caco-2	10 µM (5.79 µg/mL)	↓ *t*-BOOH-induced caspase-3	[22]
Apoptosis and proliferation/survival	Epicatechin	Caco-2	10 µM (2.9 µg/mL)	↓ *t*-BOOH-induced caspase-3	[22]
Antioxidant	Procyanidin B2	Caco-2	1–10 µM (0.6–5.79 µg/mL)	↑ GPx, ↑ GST, ↑ GR, ↑ Nrf2 translocation	[22,23]
Apoptosis and proliferation/survival	Hexamer procyanidins	Caco-2	2.5–20 µM (0.73–5.81 µg/mL)	↓ DOC-induced caspase-3, ↓ PPAR cleavage	[24]
Apoptosis and proliferation/survival	Hexamer procyanidins	Caco-2	10 µM (2.91 µg/mL)	↓ DOC-induced AKT, ERK, p38 and AP-1	[24]
Antioxidant	Hexamer procyanidins	Caco-2	2.5–20 µM (0.73–5.81 µg/mL)	*t*-BOOH-treated cells: ↓ ROS generation, ↓ LDH ↓ DOC-induced cytotoxicity, ↓ oxidant generation, ↓ NADPH oxidase, ↓ Ca^2+^	[24,25]
Anti-inflammatory	Hexamer procyanidins	Caco-2	2.5–60 µM (0.73–17.4 µg/mL)	TNF-treated cells: ↓ NF-κB activation (↓ p-IĸB, ↑ IĸB, ↓ p50 and p65 translocation, ↓ NF-κB-DNA binding), ↓ iNOS	[25]
Anti-inflammatory	Cocoa	Caco-2	50 µM (gallic acid equivalents, 14.5 µg/mL)	↓ PGE2, ↑ COX-1, IL-stimulated cells: ↓ PGE2, = IL-8, = NF-κB	[26]
Anti-inflammatory	Cocoa	Caco-2	10 µg/mL	TNF-treated cells: ↓ IL-8, ↓ COX-2, ↓ iNOS, ↓ NFκB activation	[27]
Cell cycle	Polymer procyanidins	Caco-2	5–100 µg/mL	G2/M arrest, ↓ ornithine decarboxylase, ↓ S-adenosylmethionine decarboxylase	[28]
Cell cycle	Epicatechin	LoVo	5–1000 µM (1.45–290 µg/mL)	S arrest	[29]
Apoptosis and proliferation/survival	Procyanidin B2	SW480	10–50 µM (5.79–28.93 µg/mL)	↑ proliferation, ↑ p-AKT, ↑ p-ERK	[30]
Apoptosis and proliferation/survival	Procyanidin B2	Caco-2	10–50 µM (5.79–28.93 µg/mL)	= proliferation, = p-AKT, = p-ERK	[30]
Apoptosis and proliferation/survival	Epicatechin	SW480 and Caco-2	10–50 µM (2.9–14.5 µg/mL)	= proliferation, = p-AKT, = p-ERK	[30]

^a^ Concentration in µg/mL was calculated as epicatechin equivalents.

### 2.1. Antioxidative Effects

Aerobic organisms cannot avoid free radical and ROS generation, which are produced during normal oxygen metabolism or induced by exogenous damage [9,31]. In a physiological situation, cells maintain the balance between the generation and counteraction of ROS through non-enzymatic processes (mainly glutathione (GSH)) and enzymatic defenses (catalase (CAT), glutathione peroxidase (GPx), glutathione reductase (GR), glutathione-S-transferase (GST) superoxide dismutase (SOD), *etc.*) [9,31]. However, when the cellular balance is altered and antioxidant defenses overwhelmed, cells can be damaged. Thus, ROS overproduction may lead to the formation of highly reactive oxidation products, activation of carcinogens, formation of oxidized DNA bases and DNA strand breaks. All of these modifications might provoke errors during DNA replication and genetic alterations, modulate transcription of redox-regulated proteins, *etc.*, leading to enhanced cell proliferation and tumor promotion/progression [31].

Cocoa and its flavonoids are strong antioxidant substances. Cocoa retains a potent antioxidant capacity as compared to other foods, such as teas and red wine, and this property has been associated to its flavonoid content [11]. Remarkably, the structural characteristics of flavanols allow them to act as hydrogen donors (radical-scavenging) and metal-chelating antioxidants. In addition, cocoa and its flavonoids can prevent the DNA damage caused by free radicals or carcinogenic agents acting through the modulation of enzymes related to oxidative stress (CAT, GR, GPx, SOD, *etc.*) and the alteration of the procarcinogenic metabolism by inhibiting phase I drug-metabolizing enzymes (cytochrome P450) or activating phase II conjugating-enzymes (glucuronidation, sulfation, acetylation, methylation and conjugation) [6]. In this regard, colonic cancer Caco-2 cells pretreated for 20 h with a cocoa phenolic extract or with the pure cocoa flavanols EC and PB2 at realistic concentrations (10 μg/mL for cocoa phenolic extract and 10 μM for EC and PB2, respectively) prevented acrylamide-induced cytotoxicity (5 mM for 24 h) by restraining GSH consumption and ROS generation [20]. In detail, the reduced GSH content and the subsequent enhanced ROS generation induced by the toxic were highly prevented by the cocoa extract and PB2, whereas these effects were only partially restored by EC incubation. This feature could be partially attributed to the fact that the monomer mainly acted as a free radical scavenger. Nevertheless, similar to what was reported for other polyphenols and antioxidants [6], PB2 and the cocoa phenolic extract could protect cells by counteracting several types of radicals, upregulating antioxidant defenses and modulating signaling pathways related to cell survival. In this line, PB2 and the cocoa phenolic extract were able to augment the levels of gamma-glutamyl cysteine synthase (γ-GCS) and glutathione-S-transferase (GST) in the mentioned experimental conditions in Caco-2 cells [20].

Cocoa and its phenolic compounds also exert their protective effect towards oxidative stress by targeting the transcription factor NF-E2-related factor-2 (Nrf2) and the Kelch-like ECH-associated protein 1 (Keap1), which participate in the regulation of the antioxidant response element (ARE). Thus, the regulation of Keap1 can lead to the nuclear accumulation of Nrf2 and the subsequent ARE activation [6]. Hence, catechin (100 μM) decreased lipid peroxidation and ROS and increased the activity of GPx and GR, total sulfhydryl groups, as well as the expression of Nrf2 and hemeoxygenase-1 (HO-1) in a time-dependent manner in intestinal Int-407 cells [21].

Pure PB2 and EC (1–10 μM) reduced ROS generation without affecting GSH content in Caco-2 cells, and PB2 evoked a dose-dependent increase in GPx, GR and GST activities after 20 h of incubation [22]. Consequently, Caco-2 cells treated with EC or PB2 (10 μM) for 20 h and then submitted to an oxidative stress induced by *tert*-butyl hydroperoxide (*t*-BOOH, 400 μM) presented a reduced ROS production, evoked a protection from necrosis (measured as decreased lactate dehydrogenase (LDH) leakage) and showed higher viability than cells plainly incubated with the stressor [22]. Furthermore, this protective effect exerted by PB2 (10 μM, for 20 h) against the oxidative injury induced by *t*-BOOH occurred through the upregulation of the expression of GSTP1 [23]. PB2 treatment also enhanced nuclear Nrf2 levels at 3 h, peaked at 6 h and remained elevated up to 20 h of incubation. Accordingly, this procyanidin significantly increased the GSTP1 mRNA levels and activity at 4–20 h of incubation, which was accompanied by enhanced protein expression levels at 8 and 20 h [23].

Cocoa procyanidin dimers and trimers protected Caco-2 cells from the loss of integrity of the bilayer induced by oxidants through the interaction with membrane phospholipids, presumably with their polar head group [32]. In this line, a cocoa hexameric procyanidin fraction (2.5–20 μM) interacted with the Caco-2 cell membranes preferentially at the water–lipid interface without affecting their integrity after 30 min of incubation [24,25]. Likewise, this hexameric procyanidin fraction inhibited the deoxycholic acid (DOC)-induced cytotoxicity and partly prevented the generation of oxidants following NADPH oxidase inhibition, as well as the DOC-evoked increase in cellular calcium [24,25].

### 2.2. Anti-Inflammatory Effects

Chronic inflammation, infections and immune diseases lead to oxidative stress, which has been linked to carcinogenesis [6]. In fact, chronic inflammation constitutes a major risk for colorectal cancer [6,7,33], and most inflammation-associated colorectal cancers retain the activation of the transcription factor nuclear factor-kappa B (NF-ĸB) and inflammatory mediators (tumor necrosis factor (TNFα), cyclooxygenase-(COX)-2, *etc.*), all of these markers being related to cell proliferation, anti-apoptotic activity, angiogenesis and metastasis [6].

Cocoa extract repressed the inflammatory mediator prostaglandin E2 (PGE2) in human cancer Caco-2 cells [26]. Cells treated with a polyphenolic extract of cocoa (equivalent to 50 μM of gallic acid) for 4 h and later stimulated with interleukin-(IL)-1β for 24 or 48 h displayed a reduced PGE2 synthesis, although IL-8 secretion and NF-κB activity continued to increase [26]. Unexpectedly, the cocoa polyphenolic extract and without a pro-inflammatory stimulus induced a basal PGE2 synthesis in Caco-2 cells after 24 h of incubation. This result has been related to the stimulation of COX-1, which seems to be involved in preserving the mucosal integrity [26].

Furthermore, pretreatment with a cocoa phenolic extract, at a realistic dose (10 μg/mL) for 20 h, reduced the increase in IL-8 secretion and both COX-2 and inducible nitric oxide synthase (iNOS) expressions provoked by TNFα (40 ng/mL for 24 h) in Caco-2 cells [27]. In these experimental conditions, cocoa phenolic extract reduced both phosphorylated levels of c-Jun N-terminal kinase (JNK) and nuclear translocation of NK-κB induced by TNFα, pointing to the relevance of this pathway to reduce the intestinal inflammation [27]. Similarly, Caco-2 cells treatment with 2.5–20 μM hexameric procyanidins for 30 min, prior to incubation with TNFα (10 ng/mL) for a further 5–30 min, avoided the TNFα-induced NF-ĸB activation, prevented the phosphorylation and degradation of the inhibitor of κB (IκB), p50 and RelA nuclear translocation and NF-ĸB-DNA binding, as well as iNOS expression and cell oxidant increase [25]. These effects take place because hexameric procyanidins can interact with the plasma membrane of the intestinal cells to preferentially inhibit the binding of TNFα to its receptor and the subsequent NF-ĸB activation [25]. In this regard, a very recent study on human colon cancer cells suggests that high-molecular weight polymeric cocoa procyanidins may be the most effective for preventing loss of gut barrier function and epithelial inflammation, which are critical steps in the pathogenesis of inflammatory bowel disease and colon cancer [34].

### 2.3. Antiproliferative and Apoptotic Effects

Suppression of cell proliferation and induction of differentiation and apoptosis are relevant strategies in preventive approaches. Deregulated cell cycle and resistance to apoptosis are hallmarks of cancer [6,7]. Cell cycle control is a highly regulated process, and any alteration of cell cycle-specific proteins (cyclins, cyclin-dependent kinases (CDKs), CDK inhibitors, *etc.*) can alter and/or inhibit the continuous proliferation of cancer cells [6]. Therefore, regulation of cell cycle constitutes an important target for cocoa and its polyphenols in chemoprevention.

Procyanidin-enriched extracts and procyanidins prevented Caco-2 cell growth [28]. After 48 h of treatment, procyanidins extracts with a flavanol and procyanidin content of 501 mg/g induced 25% growth inhibition, whereas the procyanidin-enriched extracts (flavanol and procyanidin content: 941 mg/g) provoked a 75% growth inhibition. In contrast, cocoa powder samples, which consisted of a flavanol and procyanidin content of 141 mg/g, did not evoke any growth inhibitory effect in Caco-2 cells. Moreover, 50 μg/mL procyanidin-enriched extracts blocked the cell cycle at G2/M phase, without inducing apoptosis, and reduced the polyamine metabolism by constraining the ornithine decarboxylase and S-adenosylmethionine decarboxylase activities, which has been partly associated with the cell cycle blockage at the G2/M phase [28]. Likewise, incubation of colon cancer-derived LoVo cells with 5 and 1000 μM EC for 24 h arrested the cell cycle in the S phase without inducing apoptosis [29]. Importantly, lower concentrations of EC seemed to slightly promote the proliferation of LoVo cells.

Programmed-cell death, which may be considered one of the important targets in a preventive approach against cancer, is a complex process that involves the active participation of affected cells in a self-destruction cascade [6,7]. Apoptosis induction implicates the participation of death receptors (extrinsic pathway) and/or the mitochondria (intrinsic route), leading to the modulation of pro- and anti-apoptotic Bcl-2 proteins (Bcl-2, Bcl-xL, Bid, Bad, Bax, *etc.*) [6,7]. Both cascades converge in a common executor mechanism involving DNA endonucleases, which cleave regulatory and structural molecules and activated proteases (caspases) and lead to the cellular death [6,7].

Cocoa polyphenols prevent the cytotoxicity evoked by oxidative stress inducers through the ability of these compounds to restrain the increase in ROS levels and the subsequent activation of caspase-3, which leads to apoptosis induction [7]. Consequently, incubation of Caco-2 cells with PB2 (10 µM) and a cocoa polyphenolic extract (10 µg/mL) or with EC or PB2 (10 μM) for 20 h protected against acrylamide- and *t*-BOOH-induced apoptosis, respectively [20,22]. Likewise, pretreatment with hexameric procyanidins (2.5–20 µM) delayed the deoxycholic acid-induced apoptosis in Caco-2 cells, as repressed caspase-3 activation by halting poly-(ADP-ribose) polymerase (PPAR) cleavage [24].

Phosphatidyl-inositol-3-kinase (PI3K)/protein kinase B (AKT and the extracellular regulated kinases/mitogen-activated protein kinase (ERKs/MAPK)) are the most relevant signaling pathways involved in cell proliferation and survival regulation [6,7]. As mentioned above, PB2 (10 µM) and a cocoa polyphenolic extract (10 µg/mL) protected colonic Caco-2 cells against acrylamide-induced cytotoxicity by avoiding the increase in the levels of p-JNK induced by the toxic, whereas ERK seemed to play an indirect protective role through the promotion of cell proliferation and survival signaling [20]. In the case of Caco-2 stressed with *t*-BOOH, PB2 (10 µM) also prevented cell damage by upregulating GSTP1 through the activation of ERK and p38 and Nrf2 translocation [23]. Similarly, preincubation of Caco-2 cells with hexameric procyanidins (10 μM, 30 min) averted oncogenic events initiated by deoxycholic acid through the interaction with cell membranes and prevented the activation of AKT, ERK and p38, as well as the transcription factor activator protein-1 (AP-1) [24]. Interestingly, PB2 (20–50 μM, 24 h) promoted cell growth in SW480 cells by enhancing p-AKT and p-ERK levels, although these proteins were unaffected under the same experimental conditions in Caco-2 cells [30]. EC (10–50 μM, 24 h) did not evoke any effect on Caco-2 and SW480 cell survival-proliferation pathways [30]. This different response, ranging from no effect of any of the flavanols on Caco-2 cells to a survival/proliferative effect of PB2 on the SW480 cell line, depends on the distinct chemical structure of the compound and the different degree of colon cancer cell differentiation [30]. Moreover, it has been suggested that the capability of procyanidins to interact with the cell membrane and to prevent cell membrane-associated events can in part explain their effects [24].

## 3. Chemopreventive Effects in Animal Models

Colon cancer prevention by cocoa and its major components can also be studied in animal models of genetic and chemically-induced colon cancer. Although colonic cell culture models have clearly demonstrated the antioxidant and chemopreventive abilities of cocoa and its flavonoids, only experimental models for colorectal cancer could offer the opportunity to assess the contribution of this natural dietary compound to the potential prevention of colon cancer. In this regard, carcinogen-induced rodent models have been shown to mimic many features of human non-familial colorectal cancer (non-genetic based) [35], which is the most common type of colon cancer and which occurs sporadically. Thus, induction of colon tumors could be achieved by the administration of carcinogens, such as nitrosamines, heterocyclic amines, aromatic amines, 1,2-dimethylhydrazine and azoxymethane (AOM). Among these, the AOM model has been extensively used to examine the chemopreventive effect of numerous compounds on colon cancer [36]. Administration of AOM to rodents induces the development of the ACF colonic preneoplastic lesions that may progress into cancer with time [1]. Indeed, ACF represent the earliest identifiable intermediate precancerous lesions during colon carcinogenesis in both laboratory animals and humans [37].

The potentially important role of cocoa and their phenolic compounds for colon cancer prevention was first proven by Weyant and coworkers [38]. In this work, the authors demonstrated that the cocoa flavanol catechin added to a diet (0.1 and 1%, which has been estimated by the authors to correspond to a daily dose of 8 mg/kg (+)-catechin in humans, that is 480 mg for a 60-kg human that might be obtained from approximately 25 g cocoa) reduced the formation of intestinal tumors by 75% and 71%, respectively, in a genetic mice model of multiple intestinal neoplasia that spontaneously develops multiple intestinal tumors. Further mechanistic studies related this effect to (+)-catechin-induced changes in integrin-mediated intestinal cell-survival signaling, involving structural alteration of the actin cytoskeleton and decreased focal adhesion kinase (FAK) tyrosine phosphorylation. All of these suggested that (+)-catechin could prevent the progression of initiated enterocytes to the adenoma stage, as FAK has been implicated in the regulation of cell migration, one of the earliest changes in adenoma development [39].

In another study, male Wistar rats were fed with a cocoa-enriched diet (12%, which according to the body surface area normalization method [40] would correspond to a daily dose of 78 g of cocoa, containing 1560 mg of polyphenols, for a 60-kg human ) for eight weeks starting two weeks before the AOM administration (20 mg/kg bw). As estimated, all rats injected with AOM developed aberrant crypts (100% incidence) [41]. Nevertheless, the cocoa-rich diet diminished the AOM-induced ACF formation and especially those ACF with larger number of crypts (≥4 crypts), which exhibit a higher tendency to progress into malignancy. Therefore, a cocoa-supplemented diet was able to abolish the early phase of chemically-induced colon carcinogenesis. This inhibitory effect of cocoa on AOM-induced preneoplastic lesions was related to its anti-oxidative and anti-inflammatory properties. Thus, cocoa circumvented the oxidative stress induced by AOM, as it recovered to control levels the reduced content of GSH and the activities of GPx, GR and GST induced by the toxic. Consequently, the cocoa-enriched diet avoided the subsequent enhancement in the phosphorylated levels of AKT and ERK provoked by AOM, as well as the decrease in the values of cyclin D1 and cell proliferation, measured as proliferating cell nuclear antigen levels (PCNA). Cocoa flavanols also repressed intestinal inflammation induced by AOM by inhibiting the NF-κB signaling and downregulating pro-inflammatory COX-2 and iNOS expression [41]. Furthermore, cocoa supplementation upregulated Bax and downregulated Bcl-xL levels in the colon tissue of AOM-treated rats, suggesting that cocoa was also able to induce apoptosis as another complementary mechanism of chemoprevention during the progression of carcinogenesis [41]. In the same rat model of AOM-induced colon cancer, a diet including dark chocolate has been reported to reduce cell proliferation and some gene expression involving inflammation (COX-2 and p-65-NF-ĸB), resulting in a lower number of early preneoplastic lesions [42].

To conclude with the animal experimentation, it has been very recently observed that dietary cocoa (5% and 10%, which according to the body surface area normalization method would approximately correspond to a daily dose of 32.5–65 g of cocoa, containing 650–1300 mg of polyphenols, for a 60-kg human) inhibits colitis-associated cancer in a mouse model of AOM/dextran sulfate sodium (DSS)-induced chronic inflammation by preserving the redox status of the animals and limiting cell proliferation [43]. Thus, cocoa ingestion for 62 days highly reduced lipid oxidation and prevented the decrease in the activities/levels of antioxidant defenses (GSH, SOD, CAT, GPx and GR) induced by AOM/DSS administration. Moreover, cocoa-rich diets effectively decreased the expression of iNOS and COX-2 and activated the Nrf2/Keap1 pathway, as well as its downstream targets, such as NQO1 and UDP-GT [43]. In the same experimental conditions, a cocoa-enriched diet was also able to suppress the IL-6/STAT3 pathway and to induce apoptosis, as increased levels of Bax and caspase-3 together with diminished Bcl-xl values were observed in cocoa-fed mice [44].

## 4. Evidence of Chemopreventive Effects in Humans

Even though cell culture and animal studies have indicated a promising cancer-preventive efficiency, chemopreventive properties of cocoa polyphenols in patients remain to be clarified. Epidemiologic studies of cocoa intake and colon cancer risk are few, and those assessing mortality by cancer provide only weak support for a benefit of cocoa. Consequently, confirmation of colon cancer-preventive efficacy in humans requires large and long-lasting controlled clinical trials.

### 4.1. Epidemiologic Studies

In support of the anti-colon cancer effect of cocoa is the case of the Kuna tribe living in the San Blas district of Panama. Kuna drink a flavanol-rich cocoa as their main beverage, providing more than 900 mg/day, and, thus, probably have the most flavonoid-rich diet of any population [45]. When cause-specific death rates from year 2000–2004 were compared in mainland Panama and the San Blas islands where Kuna live by examining death certificates, the rate of cardiovascular disease and cancer among island-dwelling Kuna was much lower than in mainland Panama. This comparatively lower risk among Kuna might reflect a very high flavanol intake [45].

Another prominent case in support of the cancer-preventive effect of cocoa in humans is the data from the Iowa Women’s Study, which provided evidence of an inverse relation between catechin consumption and colorectal cancer incidence in post-menopausal women [46]. In the same line, Rossi *et al.* [47] have shown that high dietary proanthocyanidin intake (not necessarily from cocoa) is associated with a reduced risk of colorectal cancer, particularly cancer of the rectum. Nevertheless, some human studies have failed to show a beneficial impact of cocoa intake in the rate of cancer appearance. In this line, another study did not show a significant correlation between a high chocolate dietary pattern and any stage of colorectal disease, ranging from polyps, to adenomas and colorectal cancer [48]. In addition, an adenoma study performed in North Carolina also failed to detect a significant association between chocolate consumption and lower prevalence of adenomatous polyps and colorectal cancer [49]. This lack of correlation between chocolate intake and colon cancer could be related to the intake of chocolates with a low amount of flavanols, *i.e.*, with a small percentage of cocoa, as the case of milk chocolate, and/or to a problem related to the power because of the small sample size of the group [48].

Finally, there are a number of human studies that have shown a negative effect of cocoa intake on overall cancer incidence and, in particular, on colon cancer. In a French case-control study, chocolate was identified as a risk factor for colorectal cancer [50]. In this regard, it should be highlighted that the high intake of refined carbohydrates and sugar, which is present in the chocolate, especially in those with a low amount of cocoa, has been associated with colon cancer via their effect on insulin and IGF-I [48]. In any case, the global association at least may serve to stimulate further epidemiological and experimental studies.

### 4.2. Intervention Studies

At present, there are no human intervention studies aiming to show an association between cocoa intake and colon cancer prevention, but a few human intervention trials show that cocoa positively regulates intermediary factors in cancer progression [51].

Recently, different studies have focused on the modulation of antioxidant and anti-inflammatory status by consumption of cocoa products. Spadafranca and co-workers [52] have reported that dark chocolate consumption significantly improved DNA resistance to oxidative stress. In this trial, healthy subjects were assigned to a daily intake of 45 g of dark chocolate or white chocolate (lacking flavonoids) for 14 days, and it has been shown that the oxidative damage to mononuclear blood cells DNA was diminished in the group consuming dark chocolate after 2 h of ingestion, while the effect disappeared after 22 h. The anti-inflammatory effect of cocoa flavanols has been reinforced by *in vitro* and *ex vivo* studies, although these effects have not always been replicated *in vivo* [53]. Consequently, cocoa consumption reduced NF-κB activation in peripheral blood mononuclear cells in healthy voluntaries [54], but biomarkers of inflammation, such as IL-6, were unaltered in patients at high risk of cardiovascular disease consuming cocoa powder [55]. Although the results from the limited epidemiological and human trials that are available are not conclusive, cocoa has consistently demonstrated an ability to increase serum antioxidant status and, therefore, to theoretically reduce cancer risk.

## 5. Conclusions

**Figure 2:**
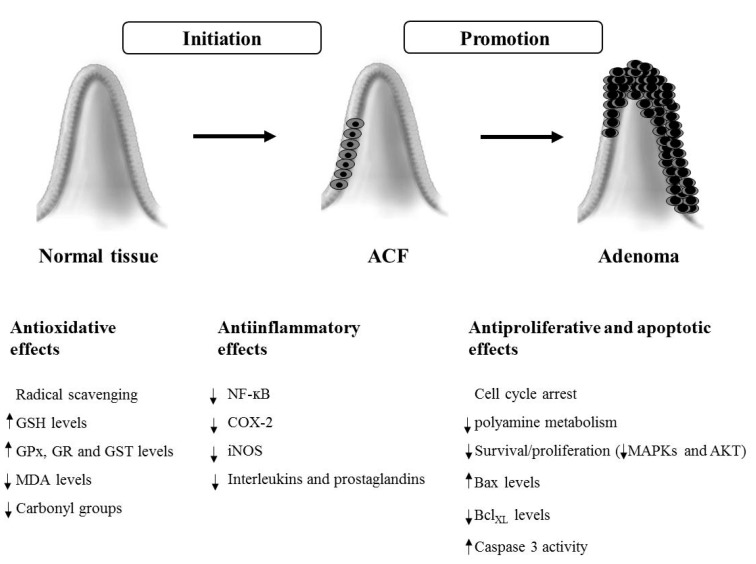
Mechanisms involved in the potential chemopreventive effects of cocoa and its flavonoids against colorectal cancer. The arrows indicate an increase (↑) or decrease (↓) in the levels or activity of the different analysed parameters. ACF, aberrant crypt foci.

This review reports the potential chemopreventive actions of cocoa and its main flavanols against colon cancer (Figure 2). In this line, their potential cellular molecular mechanisms of action include the regulation of different antioxidant defenses and numerous key proteins of signal transduction pathways related to inflammation, cell proliferation, differentiation and apoptosis. These actions could contribute to preserve a balanced redox status and to prevent the initiation and progression of an uncontrolled cell growth, as well as to avoid a pro-inflammatory environment. In addition, animal studies have proven that cocoa and its main flavanols would prevent and/or slow down the initiation promotion of colon cancer. However, amounts administrated are probably higher than what a person should normally consume and, despite these doses could be achievable through supplementation, more moderate quantities of cocoa would be desirable. Importantly, human intervention studies have reported some favorable changes in biomarkers for antioxidant status. Therefore, it could be suggested that daily consumption of small amounts of flavanols and procyanidins from cocoa or chocolate, together with an ordinary dietary intake of flavonoids, would constitute a natural approach to potentially prevent colon cancer with minimal toxicity. Nevertheless, cocoa and its derivative products merit further investigations, since the molecular mechanisms of action are not completely elucidated. Additionally, extensive well-controlled and well-designed human epidemiological and intervention studies are needed to fully assess the potential of cocoa in terms of optimal dose, route of administration, cancer targets and preventive activities.
